# Supplementary material for: Exploring the effect of microdosing psychedelics on creativity in an open-label natural setting
Source: Psychopharmacology (Berl). 2018 Oct 25;235(12):3401–13. doi: 10.1007/s00213-018-5049-7 (PMC6267140; doi:10.1007/s00213-018-5049-7)
Supplement: Supplementary file 1 — (DOCX 17 kb) [file 213_2018_5049_MOESM1_ESM.docx]

# Supplementary materials

**Article title:** Exploring the effect of microdosing psychedelics on creativity in a natural setting

**Journal name:** Psychopharmacology

**Author names:** Luisa Prochazkova*^1^, Dominique P. Lippelt*^1^, Lorenza S. Colzato^1,2,3^, Martin Kuchar^4,5^, Zsuzsika Sjoerds^1^ & Bernhard Hommel^1^

*authors contributed equally (shared first-authorship)

**Affiliations**

^1^Leiden University, Cognitive Psychology Unit & Leiden Institute for Brain and Cognition, Leiden, The Netherlands

^2^Department of Cognitive Psychology, Institute of Cognitive Neuroscience, Faculty of Psychology, Ruhr University Bochum, Bochum, Germany

^3^Institute for Sports and Sport Science, University of Kassel, Kassel, Germany

^4^Forensic Laboratory of Biologically Active Substances, Department of Chemistry of Natural Compounds, University of Chemistry and Technology Prague, Prague, Czech Republic

^5^Department of Experimental Neurobiology, National Institute of Mental Health, Klecany, Czech Republic.

**Corresponding author**
L.Prochazkova
Leiden University, Faculty of Social and Behavioural Sciences, Cognitive Psychology Unit
Wassenaarseweg 52, 2333 AK Leiden, The Netherlands
e-mail: l.prochazkova@fsw.leidenuniv.nl
Tel: +31 71 527 8085

Online Resource 1

**Additional details regarding the analyses procedure used to analyze the alkaloid content in the psychedelic truffles**

Methanol and formic acid were of LC-MS grade and were purchased from Sigma-Aldrich (Czech Republic). Ultra pure water, 18.2 MΩ-cm, was produced by a Smart2Pure 12 water purification system (Thermo Scientific, Germany).

The stock solutions of psilocin and psilocybin were prepared in methanol and the stock solutions of norbaeocystin and baeocystin in 50%(*v/v*) methanol (at a concentration 1 mg/mL and stored at -20 °C. A working solution contained a mixture of analytes and was diluted with methanol to 10 µg/mL.

***Sample preparation***

Capsules were opened and 100 mg of inner solid was weighted to dark glass tubes with screw caps. The samples were dissolved in 5 mL of methanol pre-purged with nitrogen gas. A rack with samples was subsequently covered with aluminum foil and vortexed for 150 min. The mixtures were centrifuged for 15 min at 2,000 rpm (25°C). 100 µL aliquot was diluted with 900 µL of 0.1% (*v/v*) formic acid. Samples were 100× diluted with 0.1% (*v/v*) formic acid because of high concentrations of psilocin and psilocybin. 5µL was injected into the LC-MS and samples were prepared at duplicate.

***LC conditions***

The LC system used was an Agilent 1290 Infinity (Agilent Technologies, USA) and conditions were as follows: a column Phenomenex Kinetex F5, 2.1 × 100 mm, 1.7 µm 100 Å, with pre-column, gradient elution with 0.1% (*v/v*) formic acid (mobile phase A) and methanol containing 0.1% (*v/v*) formic acid (mobile phase B) with a flow rate of 250 µL/min. The injection volume was 5µL with 3 s needle wash in a flush port. The gradient setup was: 0-5 min from 95% A to 20% A, 5-5.5 min from 20% A to 0%, 5.5-6.5 min back to 95% A and equilibration at the same level to 10 min. The valve arrangement: 0.5-6 min to the MS source.

***MS conditions***

The tandem MS used was 6460 Triple Quad LC/MS (Agilent Technologies, USA) with Jet Stream Electrospray Ionisation Source. A dynamic multiple reaction monitoring (dMRM) method was used. In brief the conditions of instrument were as follows: positive ion mode, ionisation voltage of 2300 V, a source temperature of 340°C and gas flow rate10 L/min. The values for sheath gas were 400°C and 12 L/min, and for the nebulizer 30 psi. Data was acquired and evaluated with MassHunter software (Agilent Technologies, USA).

***Alkaloid detectability and concentrations***

The evaluation of the developed analytical method (Hajkova et al. 2016) encompassed the determination of Limit of Detection (LOD) and Limit of Quantitation (LOQ), and of the applicable concentration range for each compound studied. The LOD indicates the amount of an analyte that is minimally detectable without guarantees regarding the bias or measurement errors in the results by an assay (c.q. not quantified). LOQ, on the other hand, does indicate the minimum amount of an analyte that is quantifiable. For the here described analyses LOD was determined as 3 times the ratio of signal to noise and LOQ as 10 times the ratio of signal to noise. LODs and LOQs for the alkaloids in the analyzed samples are shown in Table S1.

Table S2 contains the alkaloid concentrations for each sample type separately. The concentrations were very similar across samples for all alkaloids and the differences between estimates were lower than the estimated measurement errors for the results obtained when the data is averaged across samples. As such, in the Results we reported the concentrations for the four alkaloids collapsed across the three samples.

**Table S1** LOD and LOQ values

|  | *ng/ml* | | *µg/g (ppm)* | |
| --- | --- | --- | --- | --- |
| **Alkaloid** | **LOD** | **LOQ** | **LOD** | **LOQ** |
| Psilocybin | 0.5 | 1.0 | 0.3 | 0.5 |
| Psilocin | 1.0 | 5.0 | 0.5 | 2.5 |
| Norbaeocystin | 5.0 | 10.0 | 2.5 | 5.0 |
| Baeocystin | 1.0 | 5.0 | 0.5 | 2.5 |

^Note: Left part of the table shows final concentrations measured owing to LC-MS;
right side of table shows calculation on 1 g of capsule inner solid); LOD = Limit
of Detection; LOQ = Limit of Quantitation.^

**Table S2** Results of observed analytes for each sample type separately (µg/g [ppm])

| Alkaloid | 0.22g sample | 0.33g sample | 0.44g sample |
| --- | --- | --- | --- |
| Psilocybin | 1557 | 1595 | 1632 |
| Psilocin | 76 | 87 | 93 |
| Norbaeocystin | 9 | 8 | 8 |
| Baeocystin | 30 | 31 | 33 |
